# Optimizing imputation strategies for mass spectrometry-based proteomics considering intensity and missing value rates

**DOI:** 10.1016/j.csbj.2025.04.041

**Published:** 2025-05-03

**Authors:** Yuming Shi, Huan Zhong, Jason C. Rogalski, Leonard J. Foster

**Affiliations:** Department of Biochemistry and Molecular Biology, Michael Smith Laboratories, Life Sciences Institute, University of British Columbia, Vancouver, BC, V6T 1Z4 Canada

**Keywords:** Missing value, Missing value imputation, Proteomics, Mass spectrometry

## Abstract

Missing values (MVs) in omic datasets affect the power, accuracy, and consistency of statistical and functional analyses. In mass spectrometry (MS)-based proteomics, MVs can arise due to several reasons: peptides could be below instrumental detection limits, peptides or proteins might be absent or depleted from the sample for biological or technical reasons, or data processing could fail to detect a real signal. Several statistical and machine-learning methods have been described for imputing MVs in proteomics, such as Bayesian PCA estimation, random forest, and collaborative filtering. However, these approaches typically do not account for the underlying causes of MVs and treat all missing data uniformly, potentially introducing biases that affect the biological validity of the conclusions drawn from the imputed datasets. We found a strong negative correlation between the proportion of MVs and the average intensity for the individual protein, with more abundant proteins having fewer, but rarely zero, MVs. We divided the peptides from all proteins into nine bins based on their intensities and proportion of MV. Assuming the causes of MVs could be different in different regions, we then investigated the optimal imputation method in each bin, using normalized root mean square error (NRMSE), and found that the optimal imputation method varies across bins. A mix-imputed dataset was assembled using the optimal imputation method from each bin, and it was confirmed to exhibit low deviation from the original unimputed dataset, demonstrating mixing the optimal imputation method from each bin is a reliable strategy.

## Graphical Abstract

**Figure:**
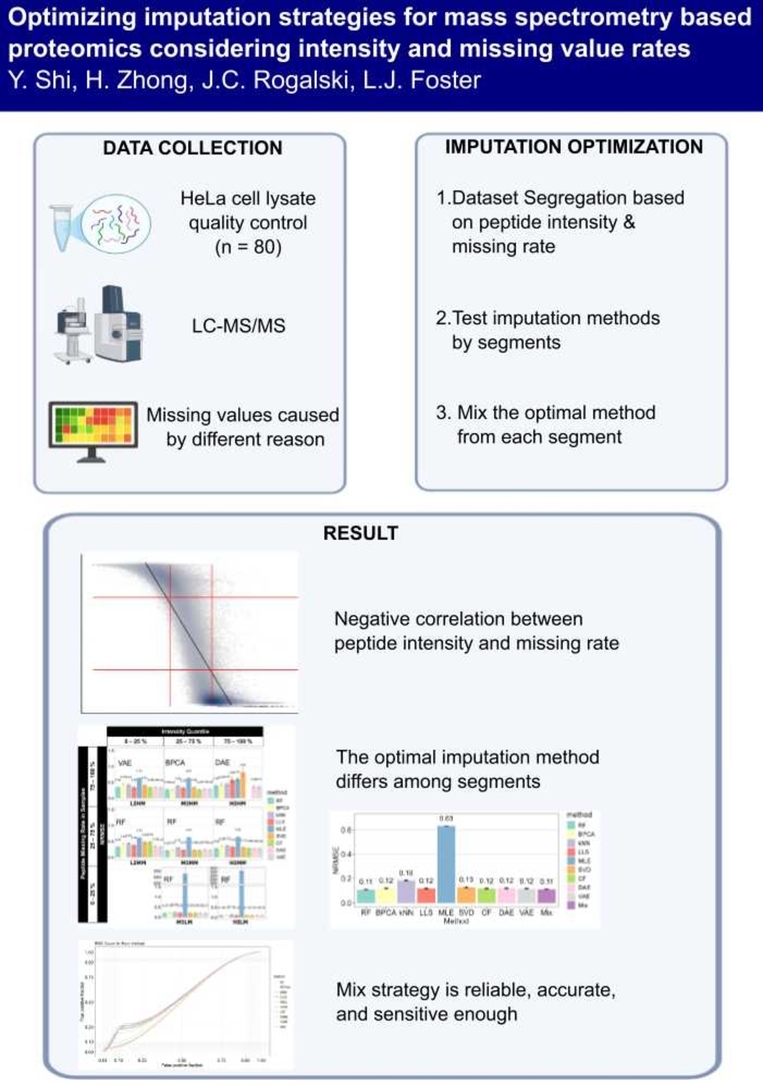

## 1 Introduction

Proteins serve as direct molecular representations of cellular activities, offering a real-time and precise depiction of phenotypic changes that surpasses the information derived from DNA sequences or mRNA expression levels. As crucial biomarkers, proteins provide insights into physiological alterations, disease mechanisms, and therapeutic targets. Mass spectrometry (MS)-based proteomics has emerged as a highly effective platform for the high-throughput quantification of peptides, proteins, and proteoforms in biological specimens. In bottom-up proteomics, proteins are enzymatically digested into peptides, which are subsequently analyzed via liquid chromatography-tandem mass spectrometry (LC-MS/MS) [1].

Despite significant advancements in MS-based proteomics, missing values (MVs) continue to present a major challenge, compromising data integrity, statistical power, and what one is able to infer about the biology of the system in question. LC-MS/MS datasets frequently contain a substantial proportion of MVs, which arise from biological variability, technical limitations, and analytical constraints [2]. Key contributing factors include detection thresholds that preclude the identification of low-abundance peptides, sample preparation inconsistencies such as protein degradation or incomplete enzymatic digestion, and database-matching failures during peptide identification. MVs are commonly categorized into two types: missing completely at random (MCAR), where absence occurs independently of peptide abundance or sample characteristics, and missing not at random (MNAR), where missingness correlates with peptide intensity, most often occurring when signals approach the instrumental detection limit [3]. However, the assumption that all MVs conform strictly to these categories can lead to erroneous imputations and introduce biases in downstream analyses.

To address MVs in MS-based proteomics, a range of statistical and machine learning-based imputation techniques have been developed. Basic approaches involve replacing MVs with zero, the mean or median of the dataset, or values drawn from a Gaussian distribution. More sophisticated methods utilize data structure and local similarity, such as k-nearest neighbor (kNN) [4] and random forest (RF) [5]. Global-structure-based techniques include singular value decomposition (SVD) [6] and Bayesian principal component analysis (BPCA) [7]. Additionally, deep learning-based approaches, including collaborative filtering (CF) [8], denoising autoencoders (DAE) [9], and variational autoencoders (VAE) [8], [10], have shown promising results in proteomics data imputation.

However, a critical limitation of existing imputation methods is their tendency to treat all missing values uniformly, disregarding the heterogeneity of missingness mechanisms. This uniform treatment can introduce biases that distort biological interpretations. Recognizing this challenge, several studies have systematically evaluated various imputation strategies. For instance, Lazar et al. [2] assessed multiple imputation approaches and emphasized the necessity of selecting strategies based on missingness patterns. Similarly, Wei et al. [11] performed a comparative analysis of imputation methods in metabolomics, recommending specific approaches tailored to distinct MV types. Kong and Wong [3] further examined missingness in proteomics, proposing a decision framework for method selection. Additionally, Shen et al. [12] conducted a comparative assessment of imputation techniques, introducing novel strategies to enhance proteomics data interpretation.

Beyond conventional methodologies, recent innovations have introduced novel imputation paradigms. Chion et al. [13] proposed a multiple imputation framework that accounts for imputation-induced variability, improving differential analysis accuracy. Medo et al. [14] introduced ProtRank, a method that circumvents imputation altogether by ranking observed protein changes, thereby mitigating bias. Moon et al. [15] developed an augmented doubly robust post-imputation inference framework, integrating machine learning models with parametric approaches to correct imputation biases and improve downstream reliability. Vanderaa and Gatto [16] revisited missing values in single-cell proteomics, evaluating the strengths and limitations of different imputation strategies. Zhou and Li [17] introduced TDimpute, a transfer learning-based neural network designed to infer missing gene expression data from DNA methylation profiles, demonstrating improved imputation accuracy. Kong et al. [3] developed ProJect, a mixed-model missing value imputation approach for proteomics data that accounts for batch effects and enhances downstream analytical outcomes.

### 1.1 Our approach and contributions

While prior studies have significantly advanced the field of missing value imputation in proteomics, our findings underscore a critical observation: missingness is highly correlated with peptide intensity. We demonstrate that low-intensity peptides exhibit a significantly higher propensity for missingness, suggesting that different mechanisms govern missingness across intensity distributions. Consequently, applying a uniform imputation strategy across all data points is inadequate.

To address this, we introduce a novel imputation framework that stratifies proteins or peptides based on intensity and missing rate, thereby enabling the selection of optimal imputation strategies for distinct categories. Our intensity-aware imputation approach enhances imputation accuracy and minimizes biases introduced by conventional methods. Validation across three independent datasets demonstrates the robustness and efficacy of our approach in real-world proteomics applications.

## 2 Material and methods

### 2.1 Datasets

We use three data-dependent acquisition (DDA) LC-MS/MS datasets, with a diversity of biological backgrounds, mass spectrometry instruments (timsTOF or Orbitrap), dataset sizes (6−93), and peptide missing percentage (22–63 %), to evaluate the performance of our missing value imputation strategy.

#### 2.1.1 Dataset A Lab Generated HeLa Cell Lysate

Dataset A is a HeLa cell lysate dataset (n = 80) of quality control runs we collected ourselves. The dataset is from the same batch of a HeLa cell lysate that was prepared and digested in bulk, from which an aliquot is analyzed at least once a day to ensure stable instrument performance. From all aliquots, 100 ng of peptides were injected and separated using NanoElute 2 UHPLC system (Bruker Daltonics) with Aurora Series Gen3 (CSI) analytical column (25 cm×75 μm 1.7 μm C18 120 Å, with CSI fitting; Ion Opticks, Parkville, Victoria, Australia). The analytical column was heated to 50 °C using a column toaster M (Bruker Daltonics). The NanoElute thermostat temperature was set at 7 °C. Buffer A consisted of 0.1 % aqueous formic acid and 0.5 % acetonitrile in water, and buffer B consisted of 0.1 % aqueous formic acid and 0.5 % water in acetonitrile. Before each run, the analytical column was conditioned with 4 column volumes of buffer A. The analysis was performed at a 0.30 μL/min flow rate. A standard 30-min gradient was run from 2 % B to 12 % B over 15 min, then to 33 % B from 15 to 30 min, then to 95 % B over 0.5 minutes, and held at 95 % B for 7.72 min.

Liquid chromatography was coupled to a Trapped Ion Mobility Spectrometry (tims) - Time of Flight mass spectrometer with dual TIMS (timsTOF Pro2; Bruker Daltonics). The Captive Spray ionization source was operated at 1800 V capillary voltage, 3 L/min drying gas, and 180°C drying temperature. During analysis, the TimsTOF Pro 2 was operated with Parallel Accumulation-Serial Fragmentation (PASEF) scan mode for DDA acquisition. The MS and MS/MS spectra were collected in positive mode, from *m/z* 100 Th to *m/z* 1700 Th, and from ion mobility range (1/ K_0_) 0.7 V·s/cm^2^ to 1.35 V·s/cm^2^. A polygon filter was applied to the mass-to-charge and the ion mobility plane to include the most likely peptide precursors and to reduce singly charged background ions.

TIMS-MS scan was set at an equal ramp time and accumulation time of 100 ms, at a rate of 9.42 Hz (100 % duty cycle). Active exclusion was enabled with a 0.4 min release and reconsidered if their intensity increased more than 4 times. For each TIMS cycle, 5 PASEF MS/MS scans were recorded (total cycle time 0.64 second). Target Intensity for parent ions was set to 10,000 cts/s with a threshold of 1000 cts/s. Isolation widths were set at 2.07 *m/z* starting at *m/z* 400 Th and 3.46 *m/z* ending at *m/z* 1000 Th. The collision energy was ramped linearly as a function of mobility value from 27 eV at 1/k0 = 0.7 V·s/cm^2^ to 55 eV at 1/k0 = 1.35 V·s/cm^2^.

All acquired data was searched using FragPipe [18] computational platform (version 20.0) with MSFragger (version 3.8), Philosopher (version 5.0), EasyPQP (version 0.1.40) against Homo sapiens database (reviewed sequences only; 20435 entries, downloaded from Uniprot on 2024/05/28). Common contaminants and decoys were added. Mass tolerance was set to −50–50 ppm for precursor and 20 ppm for fragments. Data was searched with strict trypsin protease specificity with up to 2 missed cleavages. Peptide length 7–50 amino acids, mass range *m/z* 500 Th to *m/z* 5000 Th were used. Cysteine carbamidomethylation (+57.021464) was set as a fixed modification; N-terminal acetylation (+42.0106) up to 1 and methionine oxidation (+15.994915) up to 3 and trimming of N-terminal methionine were set as variable modifications. MSBooster was enabled along with “Predict RT” and “Predict spectra”. Match between runs was enabled. Percolator was selected for PSM validation. Final reports were generated and filtered at 1 % FDR at the protein level.

From a large set of possible samples, we randomly selected 80 samples to work with here. The dataset was randomly partitioned into a training set (n = 48) and a testing set (n = 32) to test the robustness of our imputation strategy. These two datasets were imputed and tested using the same protocol in 2.2.

#### 2.1.2 Public Available Datasets

Two publicly available datasets (PXD000279 and PXD002882) acquired in DDA mode were also used for the orthogonal evaluation of our strategy. Peptide abundance matrices and sample metadata of both datasets were downloaded from PRIDE[19]. Dataset B (PXD000279) is a *Homo sapiens* and *Escherichia coli* protein mixture dataset (n = 6) [20] where *H. sapiens* protein concentrations should be identical across samples, while *E. coli* proteins are set to a ratio of 3:1 between the high- and low-dose groups. With this ground truth, we can evaluate the precision and sensitivity of imputation methods, as previously done in several proteomics missing values imputation methods evaluation studies.

Dataset C (PXD002882) is from a study of Crohn’s disease (n = 93) [21] and contains data from twenty-one Crohn’s disease patients and ten healthy individuals. The patients can be divided into three groups based on the severity of their condition, including five mild, eight moderate, and eight severe. Three samples are collected from each individual.

### 2.2 Imputation strategy

Our imputation optimization strategy can be summarized in the following steps:

1) Dataset Segregation.
  While it is difficult to know why any given value is missing, there is a strong link to the intensity of the parallel not-missing values. Therefore, we segregate datasets into sectors based on the peptide mean intensity and missing rate (high, medium, or low) across samples. Intensity thresholds are relative and dependent on the data distribution in each dataset. They are set to the 25 % and 75 % quartiles of peptide mean intensities in a dataset. For example, a peptide in high-intensity sectors typically has a mean intensity higher than 75 % of all peptides in the whole dataset. These intensity thresholds are picked because peptide intensities are normally distributed.
  Peptide MV rate thresholds are set to missing in 25 % and 75 % of the sample. For example, a peptide in the medium-missing sectors will be missing in 25–75 % of the samples. While a peptide in the low-missing sectors will be missing in less than 25 % of samples, and a peptide in the high-missing sectors will be missing in more than 75 % of the samples. The 75 % missing rate threshold is inspired by the data-trimming step of removing features missing in 75 % of the samples [8] or removing features showing in less than 4 samples [22] in published imputation methods, as we hope our method can recover as much data as possible from the missing value. The 25 % missing rate threshold is inspired by the 10–30 % missing values induced by several proteomics missing value imputation method publications [23]. Assuming a missing rate in this range would not significantly affect the nature of a dataset. Combining the four thresholds, we can divide a dataset into 9 sectors, each of which is named after four letters, such as HIHM (**H**igh **I**ntensity **H**igh **M**issing rate), MILM (**M**edium **I**ntensity **L**ow **M**issing rate), LIMM (**L**ow **I**ntensity **M**edium **M**issing rate), etc.
  For Dataset B (PXD000279, n = 6), because there are only 6 samples, a missing rate threshold at 75 % percent will only assign peptides with one valid value to the high missing subsets, making masking and downstream analysis impossible. So based on the structure of this dataset, we adjusted the missing rate thresholds to 35 % and 65 %.
2) Mask and Test.
  To evaluate our imputation strategy, we randomly remove 10 % of the abundance values in Dataset A and B as commonly done in previous studies [11], and use the original observed abundances as the ground truth for evaluating imputation performance. For Dataset C, we removed 20 % of the non-missing values to test the method in a different setting. Peptides that are missing in all samples are removed. We then impute the missing values using different methods listed in 2.3. The accuracy and sensitivity of each method are evaluated in each sector using the normalized root mean squared error (NRMSE). The method with the lowest NRMSE numeric value is identified as the optimal method for this sector.
3) Mix the Optimal.

We then assembled the whole imputed dataset (known as Mix below) by mixing the imputed subset using the optimal method from each subset. The masked whole dataset was also imputed by different imputation methods regardless of peptide intensity and missing rate. Evaluation was then conducted between the Mix and current practices (impute as a whole ignoring peptide intensity and missing rate). The Spearman’s rank correlation coefficient and the number of differentially expressed peptides were calculated for each method, and compared to the original dataset to evaluate the performance of each method.

### 2.3 Imputation methods tested

Our imputation strategy tests nine commonly used and well-examined missing value imputation methods in MS-based proteomics. We also included several newly developed deep-learning methods for testing. All these nine methods can be divided into two groups based on how they sample values to estimate missing values.

(1) *Local similarity* methods predict missing values based on similar peptides. Similarity is inferred from clustering algorithms. We selected several commonly used methods for this study, including kNN [17] and RF [10], which are reportedly performing well in previous studies.
(2) *Global structure* methods focus on the overall layout of the data, and estimate missing values from the low-dimensional representation of the proteomics abundance matrix. We picked several methods belonging to this category, including local least squares [18], maximum likelihood estimation (MLE) [17], BPCA [18], and SVD [17], as well as three deep learning methods, CF, VAE, and DAE [12].

### 2.4 Evaluation metrics

Using the masked-ground truth values (observed values O_i_) and their imputed values (simulated values S_i_), root mean square error (RMSE) is calculated sample-wise, and normalized by the standard deviation of ground-truth values to get normalized root mean square error (NRMSE).

$$\mathrm{NRMSE}=\frac{\mathrm{RMSE}}{\sigma }=\frac{\sqrt{\frac{\sum_{i=1}^{N}{\left(S_{i}-O_{i}\right)}^{2}}{N}}}{\sqrt{\frac{\sum_{i=1}^{N}{\left(O_{i}-\overset{®}{O}\right)}^{2}}{N-1}}}$$

**σ** is the standard deviation of ground-truth, $\underline{\overset{®}{O}}$ is the average log_2_-transformed peptide intensity in each sample, N is the total number of non-missing peptides in each sample.

After assembling the optimal imputed dataset with the optimal method from each sector, pair-wise Spearman’s rank correlation coefficient was calculated between the observed real values and imputed values from each method, using the stats package. We then perform differential expression analyses in Datasets B and C using the limma package [19] in R [20]. P-values are corrected by the Benjamini-Hochberg method for multiple-testing correction, peptides with p_adj_ < 0.05 are regarded as significantly differentially expressed peptides. For Dataset C, the set of significant differentially expressed peptides found on the unmasked dataset (real) is used as a reference for estimating the imputation precision, using Upset plots made by the UpsetR package [21]. Receiver operating characteristics (ROC) curves are calculated for Dataset B where all *E. coli* peptides are designed to be significantly differentially expressed (assigned as D = 1 in ROC calculation), and all human peptide abundance should be equal (assigned as D = 0 in ROC calculation). For the imputed datasets, peptides with p_adj_ < 0.05 are regarded as D = 1, while not significant peptides are regarded as D = 0.

## 3 Result

Here we utilized three datasets to develop and evaluate our imputation methods: quality control samples from our core facility (Dataset A, n = 80), a benchmarking dataset where human and *E. coli* proteins were mixed at different ratios (Dataset B, n = 6), and a real-world Crohn’s disease dataset (Dataset C, n = 92). Our approach began with an exploration of the characteristics of missing values, followed by classification and region-based optimization of imputation methods. Finally, we validated and tested the method on real-world data to ensure its robustness and applicability.

### 3.1 Method development

**Fig. 1:**
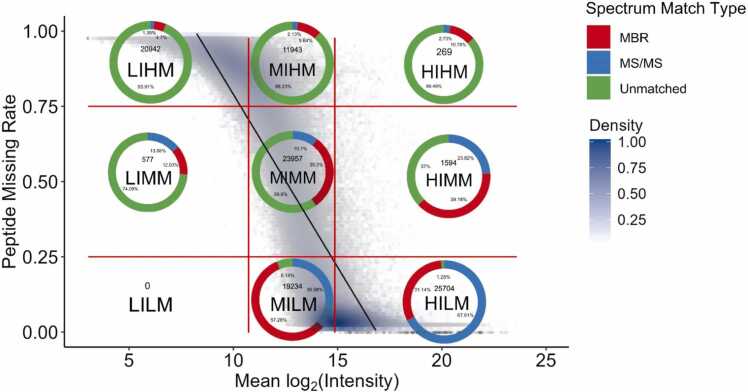
Peptides with higher average intensity have a lower chance of being missing. The x-axis is the peptide mean log₂ intensity across samples, and the y-axis is the peptide missing rate in samples. Vertical red lines mark the 25 % and 75 % mean log₂ intensity in the dataset, horizontal red lines mark the 25 % and 75 % missing rate. Each dot represents a peptide. Shades of color show the density of dots. The grey line shows the linear regression between mean log₂ intensity and missing rate. Pie charts show the proportion of spectrum match types in each sector.

We developed and optimized our imputation strategy using the quality control samples in Dataset A. We observed a strong negative correlation (R^2^ = 0.73, *p*-value < 2.2 × 10^−16^) between peptide average intensity and missing rate (Fig. 1). High-intensity peptides exhibited lower missing rates, while low-intensity peptides were more prone to missingness. This trend can also be observed when comparing the peptide spectrum match types in sectors. In the sectors with low intensity and high missing rates (e.g., LIHM and MIHM), the majority of peptides were unmatched (93.51 % and 89.22 %, respectively), meaning their intensities can neither be identified from a matching spectrum nor from match-between-runs, indicating a strong correlation between low intensity and high missingness. Conversely, in sectors with high intensity and low missingness (MILM and HILM), the proportion of unmatched peptides decreased significantly, with the unmatched rates dropping to 6.14 % and 1.25 %, respectively. This trend highlights the negative relationship between peptide average intensity and missing rate, demonstrating that peptides with greater average intensity are more likely to be consistently detected in the dataset.

**Fig. 2:**
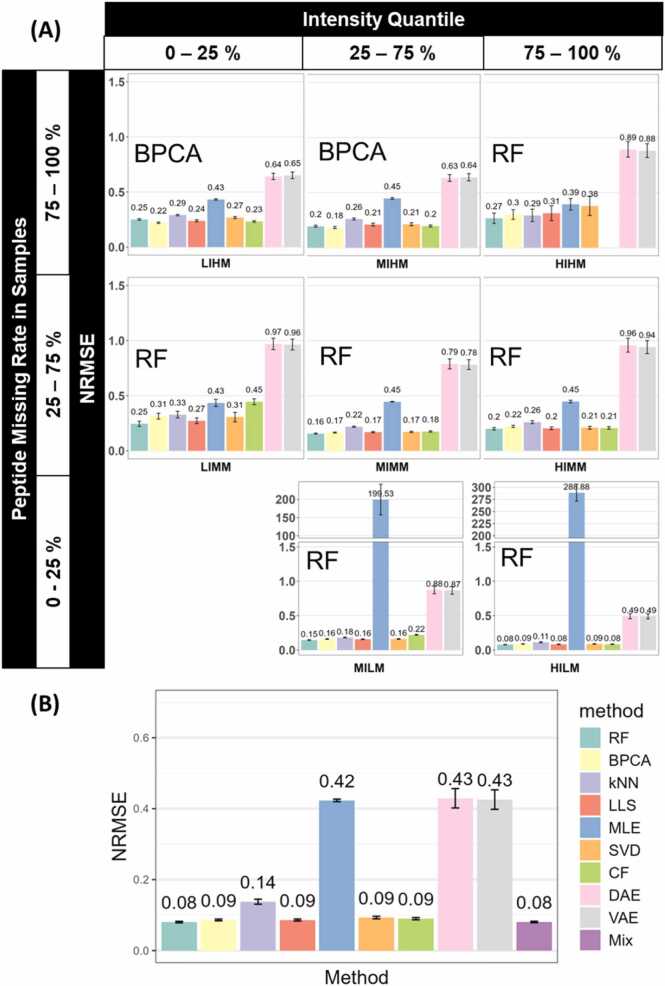
(A) The optimal imputation method differs by intensity and missing rate in Dataset A training set (n = 48). Bar plots show the average NRMSE of different methods in sectors, in the order of sector intensity quantile (top) and peptide missing rate quantile (left). Error bars represent the sample-wise 95 % confidence interval. The optimal method of each sector is annotated to the top left corner of each sector. A blank space in the figure means the corresponding method does not apply to the sector. (B) Mixing the optimal imputation methods can guarantee a low deviation in the imputed dataset. The bar plot shows the average NRMSE of imputation methods when Dataset A is imputed without segregation. Error bars represent the sample-wise 95 % confidence interval. Detailed ANOVA and Tukey's Honestly Significant Difference results are available in Supplementary Table S2, S3, and S4.

The sector difference in peptide spectrum match types also suggests peptides in different sectors are likely to be missing due to different reasons. As shown in Fig. 1, the proportion of peptides identified by MS/MS increases as the peptide missing rate decreases, with the highest proportion of MS/MS-identified peptides being found in low missing sectors (67.61 % in HILM and 36.58 % in MILM). In medium and high missing sectors, more identified peptides were found by MBR, rather than MS/MS, suggesting the “match-between-runs” (MBR) function in Fragpipe managed to salvage many potential MVs when all 80 samples were searched together. This missing rate-related difference in MS/MS-confirmed peptides suggests values could be missing for different reasons: for high-intensity peptides, it seems more likely that MVs are due to signal processing errors, and the stochastic effects of DDA analysis in precursor ion selection. While for low-intensity peptides, it seems more likely that MVs are due to the signal being too low. Therefore, given the possible variation in causes of MVs, it is necessary to impute each sector with a method that fits their source of missing values,

To find the optimal imputation method for each sector, we used NRMSE to measure how much the dataset has changed after imputation, with the method having the smallest NRMSE (For clarification, in each sector, there were several methods with low NRMSEs, and there were no significant differences between them. We picked the numerically lowest one as “the lowest”.) compared to ground truth being selected as the optimal method for a sector. The result of Dataset A training set is shown in Fig. 2(A), the optimal imputation method for peptides varies based on their intensity and missing rate. RF was the best practice in most sectors, especially in medium and high missing rate sectors. Except for MIHM and LIHM, in which BPCA stood out as the optimal method. CF was not applicable for HIHM, due to insufficient data available for the model to capture features. The difference suggests that the interaction between peptide intensity and missing rate influences imputation performance. There is no universally optimal method.

We then mixed the optimal imputed data from each sector to create the “Mix” imputed dataset. As Fig. 2(B) suggests, Mix achieves the lowest NRMSE of 0.08 in our tests, followed closely by RF with an identical NRMSE of 0.08. BPCA, LLS, CF, and SVD also have close NRMSEs of 0.09. The kNN, VAE, and DAE showed high NRMSEs of 0.42–0.43, indicating their low accuracy compared to the others. The comparably low NRMSE exhibits the accuracy of the Mix strategy in missing value imputation, emphasizing the importance of tailoring imputation methods to peptide intensity and missing rate.

The correlation analysis result in Table 1 shows that the Mix strategy, along with RF, BPCA, kNN, LLS, SVD, and CF, can preserve a strong correlation between the imputed values and the original values. This strong correlation demonstrates that the Mix strategy can well-preserve the data structure after imputation.

**Table 1:** Spearman’s rank correlation coefficients of different imputation methods.

	Method									
	Mix	RF	BPCA	kNN	LLS	MLE	SVD	CF	DAE	VAE
rho	0.997	0.996	0.995	0.989	0.996	0.896	0.995	0.996	0.890	0.891

**Fig. 3:**
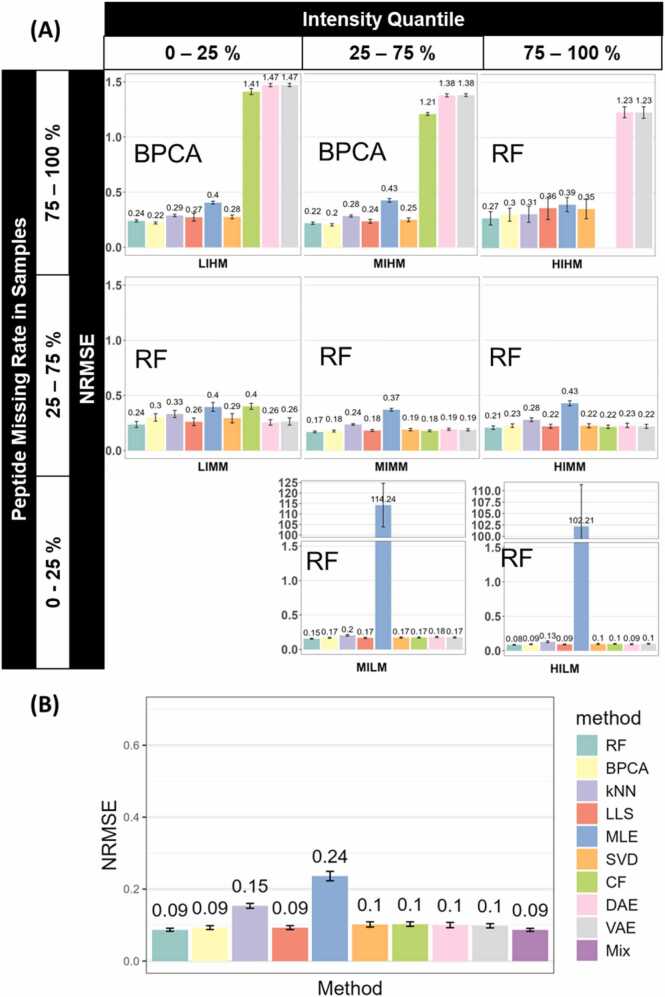
(A) The optimal imputation method differs by intensity and missing rate in Dataset A testing set (n = 32). Bar plots show the average NRMSE of different methods in sectors, in the order of sector intensity quantile (top) and peptide missing rate quantile (left). Error bars represent the sample-wise 95 % confidence interval. The optimal method of each sector is annotated to the top left corner of each sector. A blank space in the figure means the corresponding method does not apply to the sector. (B) Mixing the optimal imputation methods can guarantee a low deviation in the imputed dataset. The bar plot shows the average NRMSE of imputation methods when Dataset A testing set is imputed without segregation. Error bars represent the sample-wise 95 % confidence interval. Detailed ANOVA and Tukey's Honestly Significant Difference results are available in Supplementary Table S2, S3, and S4.

As suggested in Fig. 3(A), identical patterns were observed in the testing set. The best peptide MVs imputation method varies based on peptide intensity and missing rate. RF was the most suitable method in most sectors. While for MIHM and LIHM, BPCA was the optimal method. The Mix strategy, as shown in Fig. 3(B), has the lowest NRMSE of 0.09 in our test. Most other methods, including RF, BPCA, LLS, SVD, CF, VAE, and DAE, showed similar NRMSEs ranging from 0.09 to 0.1 The kNN and MLE methods showed high NRMSEs as in the training set, indicating their low accuracy. This resemblance between the training set and testing set demonstrated that the Mix strategy is reliable and robust for MV imputation. It is worth noticing that the deep learning method, VAE and DAE, have significantly lower NRMSEs (p < 0.05) in the testing set, despite the testing set having fewer samples. This could potentially be attributed to model underfitting and sampling bias, as the complex neural network models cannot learn enough patterns from the testing set with fewer data points, which could be less representative of the whole dataset.

### 3.2 Validation of the strategy using a benchmark dataset

**Fig. 4:**
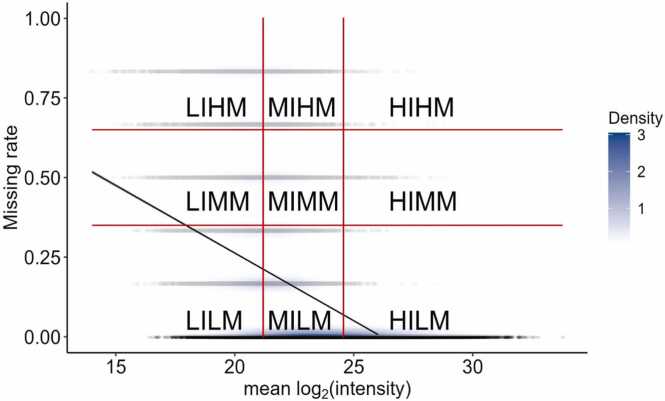
Correlation between peptide average intensity and peptide missing rate in Dataset B. The x-axis is the peptide mean log₂ intensity across samples, and the y-axis is the peptide missing rate in samples. Vertical red lines mark the 25 % and 75 % mean log₂ intensity in the dataset, horizontal red lines mark the 35 % and 65 % missing rate. Each dot represents a peptide. Shades of color show the density of dots. The grey line shows the linear regression between mean log₂ intensity and missing rate. (For interpretation of the references to color in this figure legend, the reader is referred to the web version of this article.)

We validated our imputation strategy using Dataset B, the *H. sapiens*/*E. coli* protein mixtures. The negative correlation between peptide average intensity and missing rate (R^2^ = 0.20, *p*-value < 2.2 × 10^−16^) was confirmed in the benchmark dataset as shown in Fig. 4. The density distribution map also reveals that most low-missing peptides cluster in the medium or high-intensity sector, as indicated by the dark blue area near the bottom of the plot. This pattern highlights that peptides with higher intensities are more reliably detected, whereas lower-intensity peptides are prone to miss data, consistent with typical proteomics data behavior.

**Fig. 5:**
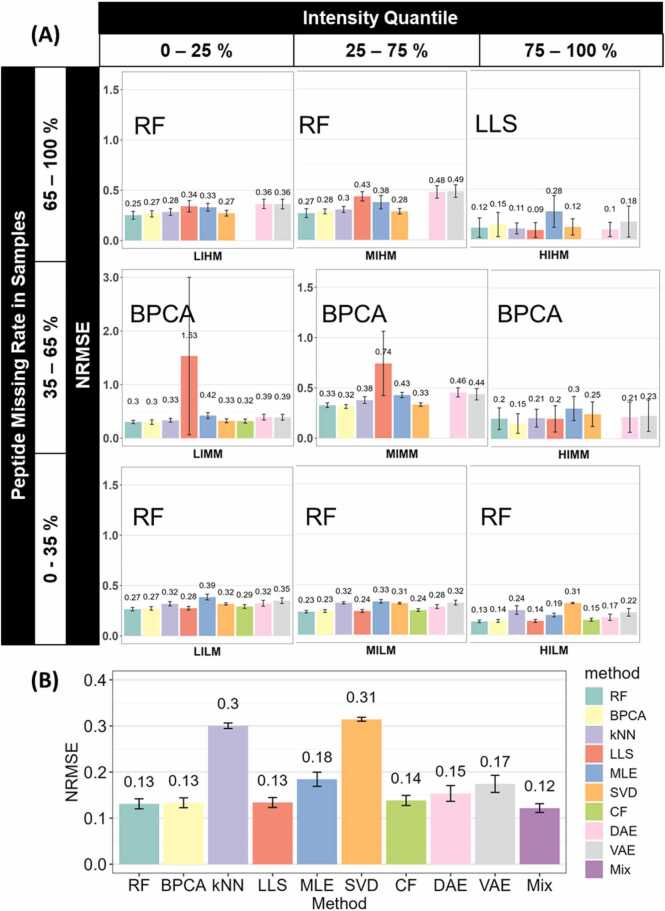
(A) NRMSE reveals the optimal imputation method from each region. Bar plots showing the average NRMSE of different methods in sectors, in the order of their corresponding intensity quantile (top) and peptide missing rate quantile (left). Error bars represent the sample-wise 95 % confidence interval. The optimal method of each sector is annotated to the top left corner of each sector. A blank space in the figure means the corresponding method does not apply to the sector. (B) The mixing strategy performed better than all other methods in Dataset B. The bar plot shows the average NRMSE of imputation methods when Dataset B is imputed with no segregation. Error bars represent the sample-wise 95 % confidence interval.

Similar to the pattern of optimal methods in Dataset A, no universally optimal imputation method was found for the validation benchmark dataset. RF was found to be the optimal imputation method in most sectors as suggested in Fig. 5(A), especially in the low-missing sectors (LILM, MILM, and HILM). In high-missing sectors (LIHM, MIHM), RF was also the optimal, except for HIHM, where LLS was the best. BPCA was the best practice in medium-missing sectors (LIMM, MIMM, HIMM). The difference in the optimal strategy in any given sector between Datasets A and B suggests the optimal imputation methods are data-dependent, further illustrating the necessity of developing a strategy to find the best imputation method for each dataset.

Consistent with Dataset A, the Mix strategy was also the top imputation method for Dataset B. As Fig. 5(B) suggests, Mix had an NRMSE of 0.12, which is the lowest among all methods. RF, BPCA, LLS, CF, and DAE all showed similar performance, with their NRMSE ranging from 0.13 to 0.15. VAE and MLE had higher NRMSE at 0.17 and 0.18. As for kNN and SVD, their NRMSE were significantly higher than others, reaching a level of 0.30–0.31. This good performance of the Mix strategy proves it is a consistently reliable method for proteomics imputation at the peptide level.

The correlation analysis results in Table 2 shows that values imputed by the Mix strategy have a strong correlation with the original values. This strong correlation demonstrates that the Mix strategy can well-preserve the data structure after imputation.

**Table 2:** Spearman’s rank correlation coefficients of different imputation methods in Dataset B.

	Method									
	Mix	RF	BPCA	kNN	LLS	MLE	SVD	CF	DAE	VAE
rho	0.993	0.991	0.991	0.954	0.991	0.983	0.949	0.990	0.988	0.984

**Fig. 6:**
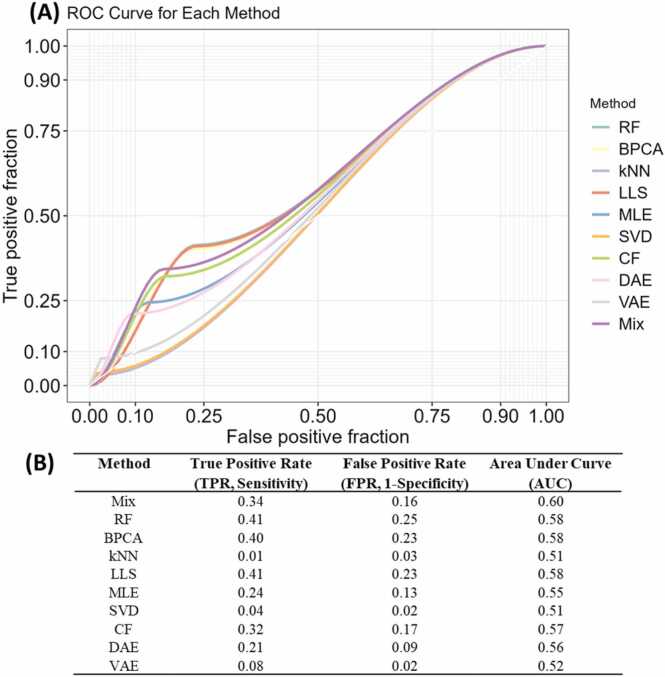
(A) ROC shows the precision (fraction of false positive on the x-axis) and sensitivity (fraction of true positive on the y-axis) of imputation methods. (B) True positive rate (TPR, 1- Specificity), false positive rate (FPR, Sensitivity), and area under curve (AUC) of each method.

Using the peptide species ground truth in Dataset B, we tested the specificity (1 - false positive rate) and sensitivity (true positive rate) of each method. Between the high and low dose group, *H. sapiens* protein abundance ratios were set to 1:1, while *E. coli* protein abundance ratios were set to 3:1. Therefore, the ground truth of this analysis is that *H. sapiens* peptides should not be differentially expressed between the two groups, while *E. coli* peptides should be differentially expressed. In that case, any significantly differentially expressed peptides found after imputation that are from *H. sapiens* are false positive results, whereas those from *E. coli* proteins are true positive results. The ROC curves in. Fig. 6(A) shows that the Mix strategy has the highest area under curve (AUC) of 0.60, followed closely by RF, BPCA, LLS, and CF, ranging from 0.57 and 0.58. As suggested in Fig. 6(B), the Mix strategy has a high sensitivity and a high specificity, the biological nature of the dataset is greatly preserved after imputation. Therefore, the Mix strategy is an ideal imputation method for missing values in MS-based proteomics.

### 3.3 Application of the imputation strategy to a real-world experiment

**Fig. 7:**
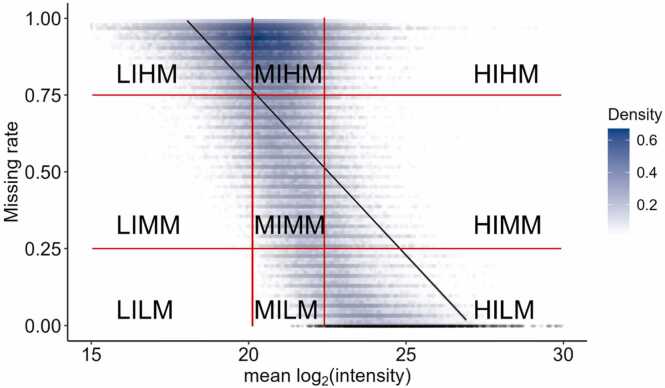
Correlation between peptide average intensity and peptide missing rate in Dataset C. The x-axis is the peptide mean log₂ intensity across samples, and the y-axis is the peptide missing rate in samples. Vertical red lines mark the 25 % and 75 % mean log₂ intensity in the dataset, horizontal red lines mark the 25 % and 75 % missing rate. Each dot represents a peptide. Shades of color show the density of dots. The grey line shows the linear regression between mean log₂ intensity and missing rate. (For interpretation of the references to color in this figure legend, the reader is referred to the web version of this article.)

Finally, we applied our imputation method to Dataset C, from a Crohn's disease study, to demonstrate our imputation optimization strategy in a practical scenario. In accordance with the pattern we observed in previous datasets, a strong negative correlation between peptide average intensity and missing rate (R^2^ = 0.41, *p*-value < 2.2 × 10^−16^) was observed (Fig. 7).

**Fig. 8:**
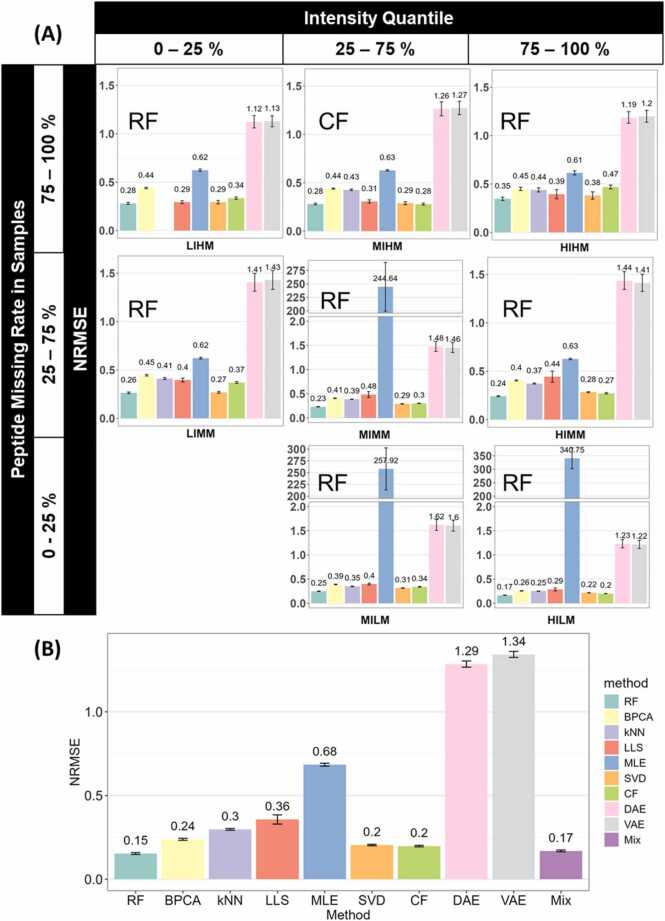
(A) The optimal imputation method from each sector for Dataset C. Bar plots showing the average NRMSE of different methods in sectors, in the order of their corresponding intensity quantile (top) and peptide missing rate quantile (left). Error bars represent the sample-wise 95 % confidence interval. The optimal method of each sector is annotated to the top left corner of each sector. A blank space in the figure means the corresponding method does not apply to the sector. (B) The mixed strategy can impute the dataset with low deviation. The bar plot shows the average NRMSE of imputation methods when Dataset C is imputed as a whole. Error bars represent the sample-wise 95 % confidence interval.

We then tested the performance of different imputation methods in each sector, as we had done for Datasets A and B (Fig. 8A). RF was predominantly the optimal method in most sectors, except for MIHM, where CF was the optimal. Autoencoder deep learning methods (VAE and DAE) consistently showed high NRMSE across sectors, suggesting they were not suitable for this dataset. This is likely attributable to the need for a large set of training data in deep learning methods, which was not met in this dataset.

When the optimal methods were mixed, we observed a similar pattern in NRMSE compared to the previous two datasets. Fig. 8(B) shows that RF and Mix are the top methods with the lowest NRMSE of 0.14 and 0.16. They were followed by CF, SVD, BPCA, kNN, LLS, and MLE, with NRMSE ranging from 0.19 to 0.64. However, the autoencoder methods, DAE and VAE showed a surprisingly high NRMSE at 1.21–1.26. Along with the low correlation coefficients of DAE and VAE shown in Table 3, this emphasizes that assessing the compatibility between methods and datasets is important to maintain data precision after any missing value imputation.

**Table 3:** Spearman’s rank correlation coefficients of different imputation methods in Dataset C.

	**Method**									
	**Mix**	**RF**	**BPCA**	**kNN**	**LLS**	**MLE**	**SVD**	**CF**	**DAE**	**VAE**
rho	0.990	0.992	0.978	0.967	0.958	0.925	0.984	0.985	0.252	0.218

**Fig. 9:**
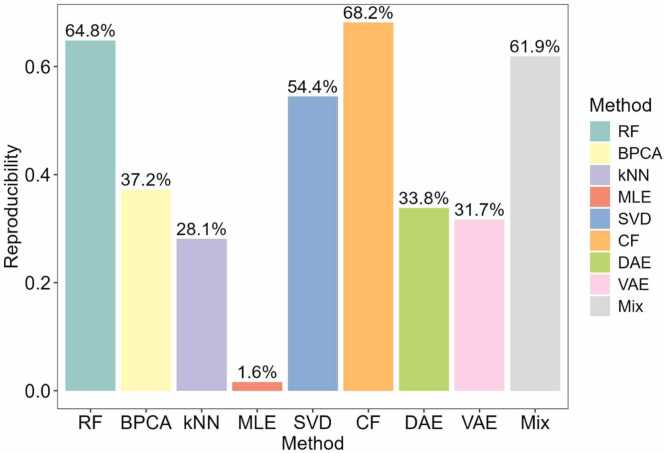
Reproducibility of significant peptides across different imputation methods in Dataset C. The bar plot shows the probability of reproducing significant peptides (y-axis) across various imputation methods (x-axis).

We further investigate the significantly differentially expressed peptide reproducibility of different imputation methods for handling MVs in proteomics data (Fig. 9). Here, reproducibility refers to the ability to consistently identify the same significantly differentially expressed peptides across datasets using different imputation methods. Among the methods, CF achieved the highest reproducibility at 68.2 %, followed closely by RF at 64.8 % and Mix at 61.9 %. Other methods such as SVD and BPCA exhibited moderate reproducibility at 54.4 % and 37.2 %, respectively. Notably, MLE performed the worst with only 1.6 % reproducibility. Meanwhile, DAE, KNN, and VAE showed reproducibility levels of 33.8 %, 28.1 %, and 31.7 %, respectively. This comparison highlights the robustness and applicability of the Mix strategy and its potential as a reliable method for MV imputation in proteomics.

## 4 Discussion & conclusions

Our study demonstrates that MVs in proteomics are strongly influenced by peptide intensity, with low-intensity peptides being more prone to missingness. Across the three datasets, we consistently observed an inverse relationship between peptide intensity and missing rate, aligning with previous findings [8]. Our 'Mix' strategy, which selects the optimal imputation method for each intensity-missing rate sector, outperforms using a single method across the entire dataset. The Mix strategy consistently achieved NRMSE, higher reproducibility of significant peptides, and greater accuracy and sensitivity in differential expression analyses across all three datasets.

The inverse relationship between intensity and missing rate can be attributed to the detection limits of mass spectrometry [3]. High-intensity peptides are more likely to be detected consistently due to their stronger signal, while low-intensity peptides are closer to the detection threshold and thus more susceptible to stochastic effects. But missingness cannot simply be a function of individual peptides being above or below the limit of detection - even high-intensity peptides can be missed, suggesting that there are multiple causes for peptides being missed. However, while intensity-dependent missingness is a critical factor, our binning approach may not capture all underlying causes of missingness. Future research should explore additional sectorization strategies, such as clustering-based methods, to better account for data heterogeneity.

Previous studies have shown that the effectiveness of imputation methods depends on composition of missingness types (missing at random or missing not at random) [24]. Plus, most studies apply a single imputation method across the entire dataset, which may introduce biases and reduce the accuracy of downstream analyses [24]. None of the exist method consider the peptide intensity-missing rate relationship we observed across datasets when imputing missing values. To address these limitations, our study systematically evaluates a wide range of imputation techniques across different intensity-missingness sectors, assuming intensity-missingness rate sectors are good representation of the nature of missingness. We propose a region-specific “Mix” imputation strategy that selects the most suitable method based on peptide intensity and missingness level, and validates this approach across multiple datasets. Our findings demonstrate that this strategy improves imputation accuracy and enhances the reproducibility of proteomics analyses. Our approach extends the existing literature by systematically evaluating a comprehensive range of imputation techniques across multiple datasets and proposing a mixing strategy that typically outperforms individual methods [8], [25]. Further functional analysis, such as Gene Ontology enrichment analysis could be applied to test the biological relevance of our strategy, as done in previous studies [22], [23]. Moreover, the improved reproducibility of significant peptides across different imputation methods underscores the reliability of our approach in biological interpretations.

Despite its robustness, the method has limitations. First, the datasets used in this study represent specific experimental conditions and may not capture the full range of variability present in proteomics data. Future research should apply this approach to diverse datasets, particularly data-independent acquisition (DIA) proteomics, where missingness patterns differ significantly from data-dependent acquisition (DDA) [22], [26], [27], [28]. Unlike DDA, where stochastic precursor selection introduces missing values, DIA reduces stochastic effects, likely lowering the overall missing rate and altering the effectiveness of imputation methods. As DIA gains popularity, understanding these differences is essential for robust imputation strategies. Second, while the Mix strategy outperformed individual imputation methods, its computational complexity remains a challenge for large-scale proteomics studies. The need to evaluate and select an optimal imputation method for each data sector introduces additional computational overhead, which may limit its scalability. Additionally, deep learning-based methods such as DAE and VAE [8] showed suboptimal performance in Dataset C, which is likely due to the small sample size. These methods typically require large training datasets to learn complex data structures effectively, and their performance may not generalize well to smaller datasets with high missingness. Future studies should explore the use of transfer learning [29] or pre-trained models [30] to mitigate this limitation. However, given the variability in imputation performance across different datasets and sectors, this computational trade-off is justified for achieving greater accuracy. With further optimizations, such as machine learning-based automation [31] and parallel computing, we can make mixed imputation strategies both computationally efficient and scalable for large datasets in the future.

In conclusion, addressing intensity-dependent missingness is crucial for robust proteomics analyses. Our findings emphasize the necessity of tailored imputation strategies over one-size-fits-all approaches. The Mix strategy offers a flexible and effective solution, improving imputation accuracy, reproducibility, and reliability in downstream analyses. By integrating diverse imputation strategies, this approach advances the robustness of proteomics research.

## Author contributions

**Y.S., H.Z.**, and **L.J.F.** conceived of the project; **J.C.R.** provided the HeLa dataset; **Y.S.** and **H.Z.** performed the data analysis and organization of the results. All the authors were involved in writing the manuscript.

## CRediT authorship contribution statement

**Jason C. Rogalski:** Validation, Methodology, Formal analysis. **Huan Zhong:** Writing – review & editing, Writing – original draft, Validation, Supervision, Software, Methodology, Formal analysis, Conceptualization. **Leonard J. Foster:** Writing – review & editing, Supervision, Resources, Project administration, Funding acquisition, Conceptualization. **Yuming Shi:** Writing – review & editing, Writing – original draft, Validation, Software, Resources, Methodology, Investigation, Formal analysis, Conceptualization.

## Declaration of Competing Interest

The authors declare no competing interests.

## Data Availability

The HeLa cell lysate dataset (Dataset A) is available at http://proteomecentral.proteomexchange.org/cgi/GetDataset?ID=PXD061701. The R and Python scripts used for data imputation and method evaluation are available at http://github.com/YumingShi25/MixImpute.

## Footnotes

Appendix A Supplementary data associated with this article can be found in the online version at doi:10.1016/j.csbj.2025.04.041.

## References

[1] Aebersold R., Mann M. (2003). Mass spectrometry-based proteomics. Nature.

[2] Lazar C., Gatto L., Ferro M., Bruley C., Burger T. (2016). Accounting for the multiple natures of missing values in label-free quantitative proteomics data sets to compare imputation strategies. J Proteome Res.

[3] Kong W. (2023). ProJect: a powerful mixed-model missing value imputation method. Brief Bioinform..

[4] Troyanskaya O. (2001). Missing value estimation methods for DNA microarrays. Bioinformatics.

[5] Stekhoven D., Bühlmann P. (2012). MissForest-non-parametric missing value imputation for mixed-type data. Bioinformatics.

[6] Alter O., Brown P., Botstein D. (2000). Singular value decomposition for genome-wide expression data processing and modeling. Proc Natl Acad Sci USA.

[7] Oba S., Sato M., Takemasa I., Monden M., Matsubara K., Ishii S. (2003). A Bayesian missing value estimation method for gene expression profile data. BIOINFORMATICS.

[8] Webel H. (2024). Imputation of label-free quantitative mass spectrometry-based proteomics data using self-supervised deep learning. Nat Commun.

[9] Gondara L., Wang K., Phung D., Tseng V.S., Webb G.I., Ho B., Ganji M., Rashidi L. (2018). Advances in knowledge discovery and data mining.

[10] Nazábal A., Olmos P., Ghahramani Z., Valera I. (2020). Handling incomplete heterogeneous data using VAEs. Pattern Recognit.

[11] Wei R. (2018). Missing value imputation approach for mass spectrometry-based metabolomics data. Sci Rep.

[12] Shen M. (2022). Comparative assessment and novel strategy on methods for imputing proteomics data. Sci Rep.

[13] Chion M., Carapito C., Bertrand F. (2022). Accounting for multiple imputation-induced variability for differential analysis in mass spectrometry-based label-free quantitative proteomics. PLoS Comput Biol.

[14] Medo M., Aebersold D., Medová M. (2019). ProtRank: bypassing the imputation of missing values in differential expression analysis of proteomic data. BMC Bioinform..

[15] Moon H., Du J.-H., Lei J., Roeder K. (2024). Augmented doubly robust post-imputation inference for proteomic data. ArXiv Prepr ArXiv.

[16] Vanderaa C., Gatto L. (2023). Revisiting the thorny issue of missing values in single-cell proteomics. J Proteome Res.

[17] Zhou X., Chai H., Zhao H., Luo C., Yang Y. (2020). Imputing missing RNA-sequencing data from DNA methylation by using a transfer learning-based neural network. Gigascience.

[18] Kong A.T., Leprevost F.V., Avtonomov D.M., Mellacheruvu D., Nesvizhskii A.I. (2017). MSFragger: ultrafast and comprehensive peptide identification in mass spectrometry–based proteomics. Nat Methods.

[19] Perez-Riverol Y. (2022). The PRIDE database resources in 2022: a hub for mass spectrometry-based proteomics evidences. Nucleic Acids Res.

[20] Cox J., Hein M.Y., Luber C.A., Paron I., Nagaraj N., Mann M. (2014). Accurate proteome-wide label-free quantification by delayed normalization and maximal peptide ratio extraction, termed MaxLFQ. Mol Cell Proteom.

[21] Mottawea W. (2016). Altered intestinal microbiota–host mitochondria crosstalk in new onset Crohn’s disease. Nat Commun.

[22] Harris L., Fondrie W.E., Oh S., Noble W.S. (2023). Evaluating proteomics imputation methods with improved criteria. J Proteome Res.

[23] Jin L. (2021). A comparative study of evaluating missing value imputation methods in label-free proteomics. Sci Rep.

[24] Dabke K., Kreimer S., Jones M.R., Parker S.J. (2021). A simple optimization workflow to enable precise and accurate imputation of missing values in proteomic data sets. J Proteome Res.

[25] Kong W., Hui H.W.H., Peng H., Goh W.W.B. (2022). Dealing with missing values in proteomics data. Proteomics.

[26] Hediyeh-Zadeh S., Webb A.I., Davis M.J. (2023). MsImpute: estimation of missing peptide intensity data in label-free quantitative mass spectrometry. Mol Cell Proteom.

[27] Fröhlich K. (2024). Data-independent acquisition: a milestone and prospect in clinical mass spectrometry–based proteomics. Mol Cell Proteom.

[28] Liu M., Dongre A. (2021). Proper imputation of missing values in proteomics datasets for differential expression analysis. Brief Bioinform.

[29] Yang J., Chen W., Xiao X., Zhang Z. (2025). Explainable deep learning method for power system stability evaluation with incomplete voltage data based on transfer learning. Measurement.

[30] Hayat A., Hasan M.R. (2025). A context-aware approach for enhancing data imputation with pre-trained language models. Presente Proc 31st Int Conf Comput Linguist.

[31] Yusof Z.B. (2025). Integrating artificial intelligence in big data analytics: a framework for automated data processing and insight generation. Orient J Emerg Paradig Artif Intell Auton Syst.

