# Supplementary material for: Optimizing imputation strategies for mass spectrometry-based proteomics considering intensity and missing value rates
Source: Comput Struct Biotechnol J. 2025 May 3;27:1818–26. doi: 10.1016/j.csbj.2025.04.041 (PMC12136770; doi:10.1016/j.csbj.2025.04.041)
Supplement: Supplementary file 1 — Supplementary material [file mmc1.docx]

#### **Appendices**

Table S1 ANOVA Result of NRMSEs for each sector in Dataset A

| Dataset | Region | Degree of Freedom | Sum of Squares | Mean of Squares | F value | p-value |
| --- | --- | --- | --- | --- | --- | --- |
| Training Set | HIHM | 7 | 23.224 | 3.318 | 68.316 | 1.464×10^-62^ |
|  | HIMM | 8 | 38.853 | 4.857 | 392.823 | 9.444×10^-185^ |
|  | HILM | 8 | 3555930.089 | 444491.261 | 1052.579 | 8.038×10^-274^ |
|  | MIHM | 8 | 13.918 | 1.740 | 448.917 | 6.850×10^-200^ |
|  | MIMM | 8 | 27.109 | 3.389 | 543.527 | 6.232×10^-210^ |
|  | MILM | 8 | 1692738.611 | 211592.326 | 85.668 | 1.288×10^-83^ |
|  | LIHM | 8 | 11.701 | 1.463 | 500.715 | 8.361×10^-209^ |
|  | LIMM | 8 | 31.070 | 3.884 | 237.121 | 7.474×10^-147^ |
| Training Set | HIHM | 7 | 38.904 | 5.558 | 134.731 | 6.361×10^-80^ |
|  | HIMM | 8 | 1.028 | 0.128 | 46.679 | 7.896×10^-47^ |
|  | HILM | 8 | 296572.704 | 37071.588 | 481.053 | 2.281×10^-158^ |
|  | MIHM | 8 | 72.336 | 9.042 | 5545.09 | 3.129×10^-297^ |
|  | MIMM | 8 | 0.841 | 0.105 | 149.036 | 8.957×10^-95^ |
|  | MILM | 8 | 370070.881 | 46258.86 | 465.208 | 1.758×10^-156^ |
|  | LIHM | 8 | 87.684 | 10.96 | 3746.162 | 5.817×10^-275^ |
|  | LIMM | 8 | 0.868 | 0.108 | 11.895 | 1.698×10^-14^ |

Table S2 NRMSE values in 4 digits from Fig 2(B) and Fig 3(B).

| Dataset | Method | NRMSE mean | NRMSE standard error |
| --- | --- | --- | --- |
| Training set | Mix | 0.0867 | 0.0021 |
|  | RF | 0.0870 | 0.0022 |
|  | BPCA | 0.0930 | 0.0027 |
|  | kNN | 0.1535 | 0.0035 |
|  | LLS | 0.0930 | 0.0027 |
|  | MLE | 0.2362 | 0.0067 |
|  | SVD | 0.1018 | 0.0037 |
|  | CF | 0.1028 | 0.0032 |
|  | DAE | 0.1001 | 0.0037 |
|  | VAE | 0.0981 | 0.0030 |

Table S3 ANOVA result of imputation methods in the training set and testing set.

| Dataset |  | Degree of Freedom | Sum of Squares | Mean of Squares | F value | p-value |
| --- | --- | --- | --- | --- | --- | --- |
| Training Set | Method | 9 | 10.954 | 1.217 | 597.162 | 8.99×10^-249^ |
|  | Residuals | 464 | 0.946 | 0.002 |  |  |
| Testing Set | Method | 9 | 0.611 | 0.068 | 167.622 | 2.77×10^-113^ |
|  | Residuals | 309 | 0.125 | 4.05×10^-249^ |  |  |

Table S4 Tukey test result between the Mix strategy and other methods in Dataset A training and testing sets.

| Dataset | Method Comparison | Difference | Lower Bound of Confidence Interval | Upper Bound of Confidence Interval | Adjusted p-value |
| --- | --- | --- | --- | --- | --- |
| Training Set | MIX-BPCA | -0.006 | -0.035 | 0.024 | 1.000 |
|  | MIX-KNN | -0.057 | -0.086 | -0.028 | 5.50×10^-8^ |
|  | MIX-LLS | -0.006 | -0.035 | 0.024 | 1.000 |
|  | MIX-MLE | -0.343 | -0.373 | -0.313 | 9.00×10^-12^ |
|  | MIX-SVD | -0.013 | -0.042 | 0.017 | 0.939 |
|  | MIX-RF | 3.05×10^-4^ | -0.029 | 0.030 | 1.000 |
|  | MIX-CF | -0.009 | -0.039 | 0.020 | 0.991 |
|  | MIX-DAE | -0.349 | -0.378 | -0.320 | 9.00×10^-12^ |
|  | MIX-VAE | -0.345 | -0.374 | -0.316 | 9.00×10^-12^ |
| Testing Set | MIX-BPCA | -0.006 | -0.022 | 0.010 | 0.963 |
|  | MIX-KNN | -0.067 | -0.083 | -0.051 | 1.01×10^-12^ |
|  | MIX-LLS | -0.006 | -0.022 | 0.010 | 0.963 |
|  | MIX-MLE | -0.150 | -0.166 | -0.133 | 1.01×10^-12^ |
|  | MIX-SVD | -0.015 | -0.031 | 0.001 | 0.085 |
|  | MIX-RF | -3.05×10^-4^ | -0.016 | 0.016 | 1.000 |
|  | MIX-CF | -0.016 | -0.032 | -4.12×10^-5^ | 0.049 |
|  | MIX-DAE | -0.013 | -0.029 | 0.003 | 0.197 |
|  | MIX-VAE | -0.011 | -0.027 | 0.005 | 0.415 |

The UpSet plot displays the number of significantly differentially expressed peptides that overlap between imputation methods. The bars on the bottom left indicate the total number of significantly differentially expressed peptides in each method, and the intersections to the right show the number of peptides shared across different methods.


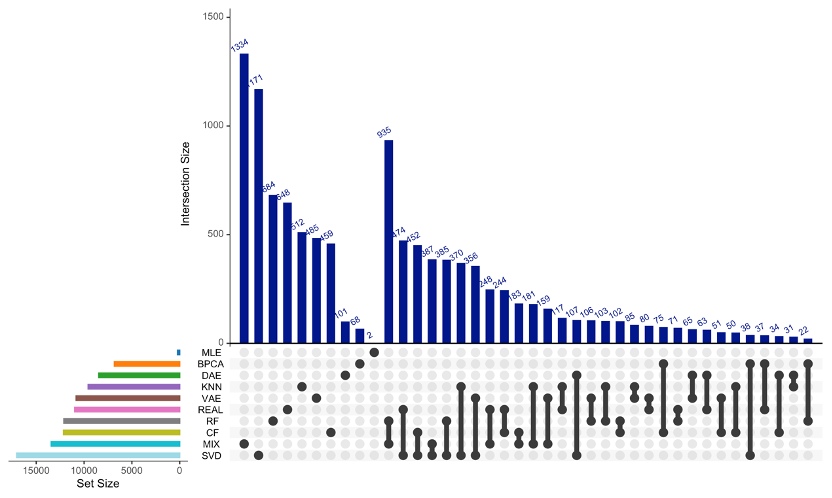


**Fig. S1 UpSet plot showing the intersections of significantly differentially expressed peptides from various imputation methods in Dataset C.**

The heatmap represents the log_2_fold-change (logFC) of peptides across various imputation methods. Each column corresponds to an imputation method, and each row represents a peptide. *Real* is the dataset prior to value masking and imputation. Blank spaces represent missing values. The color scale ranges from blue (indicating negative logFC values, which suggest downregulation) to red (indicating positive logFC values, which suggest upregulation). The intensity of the color reflects the magnitude of the logFC, with darker blue and red shades indicating stronger downregulation and upregulation, respectively. The peptides are clustered based on their expression profiles across the imputation methods using hierarchical clustering with Euclidean distance and complete linkage.


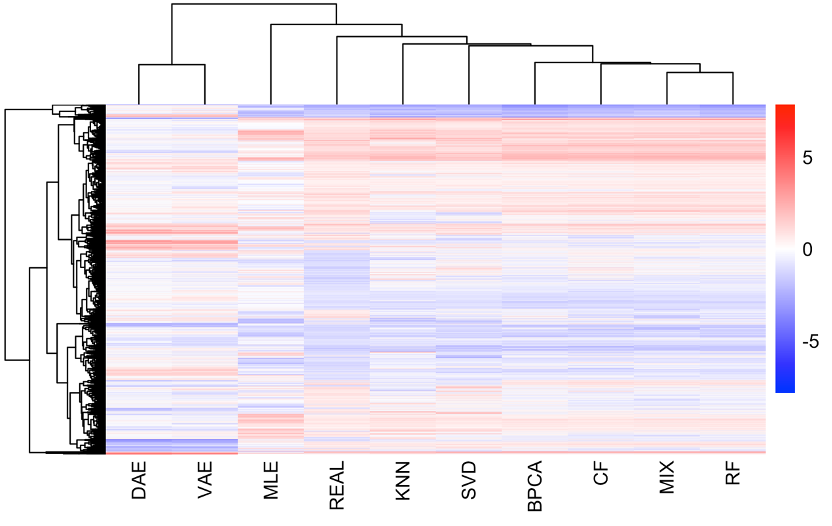


**Fig. S2 Heatmap of logFC values for different imputation methods in dataset C.**
